# Annotations

**Published:** 1887-11-05

**Authors:** 


### ANNOTATIONS.

**"Go thou and do likewise."** THERE are in England, and we hope in other countries, a great many men and women of all ranks who are worthy of the most honoured name with which the world is acquainted—the name of "Christian." The Rev. G. J. Valentine, vicar of Holme Eden, and his wife, have done, and are still doing, deeds of self-denial worthy of the rarest periods of knightly chivalry or of religious enthusiasm. The simple story is the best presentation of an example which all men will view with sympathy and reverence. As is well known to readers of the daily newspapers, there has been an outbreak of typhoid, scarlet fever, and measles in the village of Burnriggs, in Cumberland, of a most alarming and dangerous character. The sanitary conditions of the village have for years been utterly deplorable, and an unusually large proportion of the inhabitants have been struck down by violent disease. From the first Mr. and Mrs. Valentine have set themselves to be the responsible father and mother of their poor parishioners. They have visited them daily, raised funds for providing them with suitable food and trained nursing, secured isolation wherever possible, and promptly set to work to remove all unsanitary conditions without delay. In these truly parental duties they have been ably and unweariedly assisted by two ladies of position, and others in the neighbourhood. Their energetic measures have been rewarded with success, and the dangerous epidemic is rapidly subsiding. A crowning instance of devotion completes the ideal story. The Vicar, during the progress of the plague, was offered a living in the south, with which he was much pleased, and which he intended to accept. He was informed that his acceptance must be immediate, or not at all. Whereupon he decided that he could by no means leave his poor parishioners until they were restored to health and prosperity. He still remains their pastor and their friend. No commentary can add anything to this simple story. Each of us may say to himself: "Should I have done the like in like circumstances?"

**Intelligent Reciprocity.** Experienced persons on visiting strange hospitals for the first time, are frequently moved to astonishment at the peculiar character of the structures which are called by the name and used for the purposes of hospitals. It is too often the fact that of the intelligent adaptation of means to ends, hospital buildings may be cited as examples of conspicuous failure. This is a circumstance easy of explanation among those who know, though the explanation is anything rather than a justification. Hospitals have for the most part, and very properly been built by architects; but in many instances architects who have preferred to walk by the candle-light of their own predilections, rather than by the illumination to be gained from known facts, and from persons taught by daily experience to know the details of hospital requirements. An architect may be highly skilled in the general principles of construction, and yet have little knowledge of the special features of such complicated buildings as hospitals and asylums, which differ in a thousand important particulars from edifices of every other class. Architects cannot be blamed for this; but they can and should be blamed very severely if they fail to avail themselves of the knowledge of experts in hospital administration, when such knowledge is easily available. The man who from personal vanity refuses to ask for information of which he is ignorant, and yet which it is of the first importance for the efficiency of his work that he should know, is not merely a fool—he is a vicious person who chooses that other people should suffer from his want of knowledge, rather than that he himself should appear to be less than infallible. A week or two ago Mr. Burdett—who, whatever else he may be ignorant of, at least possesses an absolutely unique knowledge of hospitals, their structure, requirements, and administration—addressed, by invitation, the assembly gathered together at the opening of the Hastings Hospital. A report of the address, which dealt largely with those very details with which experience alone can make a man familiar, was printed, and as this journal had not space for it at the moment, copies were sent to about twenty different journals interested in architecture. In every case but one these were most courteously received and extensively made use of. The Editor of the *Builder* was a notable exception to this general journalistic courtesy. With a graceful dignity worthy of the magnificent Dr. Fillgrave, who was conscious that he, at any rate, had nothing more to learn either of science or of practice, this enlightened editor, who is presumably an architect, returned the copy of Mr. Burdett's address, with the following polite note: "I am obliged to you for the enclosed, but I do not attach much value to Mr. Burdett's opinions." Of course, Mr. Burdett, when he reads this, will feel duly extinguished. That, however, is a small matter. What we wish to point out is that if builders and architects hold themselves to be so absolutely infallible in their own judgment as totally to refuse either hints or instruction from those whose lives have been spent in finding out, often by painful experience, the defects of hospital buildings as frequently constructed, there is little hope of scientific and practical improvement in that most important class of buildings. Doctors and hospital managers may well stand aghast at this practical repudiation of their experienced aid.

**Farcical Inquests.** An inquest is supposed to be an inquiry into the causes and circumstances of sudden deaths, with a view to eliciting whether they are due to accident, natural causes, suicide, or murder. A recently-received communication from a medical correspondent seems to prove that these desirable ends are not always attained. He states that he was sent for at 1.45 a.m. to see the body of a man, who was avowedly already dead, at a lodging-house in one of the poor East-end localities. On being ushered into the room where the corpse was lying—and in which several other men were sleeping—he found it to be that of a well-built man of middle age, with no indication upon it to show the cause of death. He collected all the information he could obtain, and applied to the coroner for leave to make a post-mortem examination. The request was refused, and the inquest was ordered to take place two days later. The facts there brought out were to the effect that the deceased had left the workhouse the morning preceding his death apparently in fair health; that he had not asked to see the doctor, nor complained of suffering in any way. At ten p.m. he was sitting in the lodging-house kitchen with a number of other men, to some of whom he was well known, conversing with them and giving no reason for anyone to suppose he was unwell. At half-past ten he went to bed and apparently to sleep, but about one a.m. he sat up, complained of severe pain in the heart (or stomach?), and died shortly afterward. One witness stated that the deceased had complained of a cough; and on this evidence the coroner asked our correspondent to name the cause of death. As might have been expected, he declined to give an opinion on such insufficient data; on which the coroner said to him, "Your evidence, sir, is absolutely useless, and I must give the jury my opinion as to the cause of death." The coroner, it is hardly necessary to state, has no medical qualification whatever. After much discussion, the doctor was got to admit that death might have been due to syncope. The coroner, forgetting, or not knowing, that syncope is a symptom and not a disease, ordered the body to be buried forthwith. Our correspondent also sends us a newspaper cutting giving an account of an inquiry held by Mr. Wynne E. Baxter regarding a similar case of sudden death. The only medical evidence was that of a surgeon, who had not seen the deceased till life was extinct. He said that the body was that of a well-nourished man, who might possibly have a weak heart; and on the strength of this statement a verdict of death from heart disease was returned. It is clear that in either of these cases death might have been due to poison, but the inquiries were so incomplete that no definite opinion can justly be given. Such inquests as these are disgraceful, and by the indifference displayed to the elucidation of truth, tend to the encouragement of crime.

